# Subjectivity of the Anomalous Sense of Self Is Represented in Gray Matter Volume in the Brain

**DOI:** 10.3389/fnhum.2017.00232

**Published:** 2017-05-09

**Authors:** Noriaki Kanayama, Tomohisa Asai, Takashi Nakao, Kai Makita, Ryutaro Kozuma, Takuto Uyama, Toshiyuki Yamane, Hiroshi Kadota, Shigeto Yamawaki

**Affiliations:** ^1^Department of Psychiatry and Neurosciences, Institute of Biomedical and Health Sciences, Hiroshima UniversityHiroshima, Japan; ^2^Center of KANSEI Innovation, Hiroshima UniversityHiroshima, Japan; ^3^Nippon Telegraph and Telephone Communication Science Laboratories, Human Information Science LaboratoryKanagawa, Japan; ^4^Department of Psychology, Graduate School of Education, Hiroshima UniversityHiroshima, Japan; ^5^Faculty of Medicine, Hiroshima UniversityHiroshima, Japan; ^6^Research Institute, Kochi University of TechnologyKochi, Japan

**Keywords:** voxel-based morphometry, minimal self, ownership, agency, narrative self

## Abstract

The self includes complicated and heterogeneous functions. Researchers have divided the self into three distinct functions called “agency,” “ownership,” and “narrative self”. These correspond to psychiatric symptoms, behavioral characteristics and neural responses, but their relationship with brain structure is unclear. This study examined the relationship between the subjectivity of self-related malfunctions and brain structure in terms of gray matter (GM) volume in 96 healthy people. They completed a recently developed self-reported questionnaire called the Embodied Sense of Self Scale (ESSS) that measures self-related malfunctions. The ESSS has three subscales reflecting the three distinct functions of the self. We also determined the participants’ brain structures using magnetic resonance imaging (MRI) and voxel-based morphometry (VBM). Multiple regression analysis revealed a significant negative correlation between ownership malfunction and the insular cortex GM volume. A relationship with brain structure could thus only be confirmed for the ESSS “ownership” subscale. This finding suggests that distinct brain structures feel ownership and that the ESSS could partly screen for distinct brain structures.

## Introduction

For centuries, researchers have searched for the “self” (consciousness) in the brain, but no specific region seems to be dedicated to this (Legrand and Ruby, 2009). This is probably because the entire brain is involved in multiple functions and works as a network, forming what is called the default mode network (Northoff et al., 2006; Grimm et al., 2011; Qin and Northoff, 2011; Lipsman et al., 2014). The self can be regarded as surveilling the body, actions, and even the external environment (i.e., perception), which suggests that activity and functions corresponding to the self are distributed throughout the brain.

To better understand the self and its neural correlates, researchers have divided the self into essential and distinct functions. For example, Gallagher (2000) has postulated two components of the self: the minimal self and the narrative self. The minimal self is the online sensation of self and includes the sense of body (i.e., ownership) and action (i.e., agency). The narrative self is the offline storage for maintaining the sense of past and future self and includes autobiographical memory, personality and identity.

This categorization is still used in many studies because these concepts can be studied and, more importantly, tested using cognitive or neuroscientific methodologies. These functional selves have been examined theoretically through philosophy, clinical investigations and even computer models; subjectively through questionnaires and interviews; behaviorally through observations and experiments; electro-physiologically through electroencephalography (EEG) and skin conductance responses; and neurologically through functional and structural brain imaging. For example, the rubber hand illusion modulates our sense of ownership of our hand (Botvinick and Cohen, 1998). This has been examined using behavioral responses (Pavani et al., 2000), skin conductance responses (Armel and Ramachandran, 2003), skin temperature (Moseley et al., 2008), EEG (Kanayama et al., 2007, 2017; Press et al., 2008; Evans and Blanke, 2013), functional magnetic resonance imaging (fMRI; Ehrsson et al., 2004; Tsakiris et al., 2010; Brozzoli et al., 2012), and Bayesian causal modeling (Samad et al., 2015). The relationship between ownership and agency has also been experimentally investigated (Kalckert and Ehrsson, 2012, 2014). As a result, we now know that in healthy people, the subjectively reported minimal and narrative selves are expressed through behavior, physiological responses and brain activity.

However, previous studies have produced inconsistent results even when using the same measurements. This suggests the existence of individual differences in consciousness of the self. Traditional psychological studies have repeatedly shown the impact of individual differences using validated questionnaires (for schizophrenia, see Asai et al., 2011; Kanayama et al., 2009 for depersonalization; and Kanayama et al., 2008 for dissociative disorder). In cognitive neuroscience, some recent studies have shown that neural responses may be modulated by cortical structure (Suzuki et al., 2013), spontaneous cortical activation (Northoff et al., 2010; Nakao et al., 2013), and their interaction (Tavor et al., 2016), suggesting individual differences in neural responses as well. For a deeper understanding of the functional self, including the individual differences found in cognitive neuroscience studies, it is important to elucidate the relationship between individual differences measured using subjective reports and those measured neurologically. Some studies have shown that experience and learning induce structural changes in the human brain (Draganski and May, 2008; May, 2011). It may therefore be informative to compare anatomical brain structure with individual differences in the subjectively reported functional self. However, the relationship between anatomical brain structure and subjectivity of the functional self remains unclear. While some neuropsychological and psychiatric studies of patients with schizophrenia or brain lesions have investigated this, they did not measure subjectivity of the functional self in healthy subjects in daily life (rather than during a specific task) as an individual difference variable.

A previous study that applied exploratory factor analysis to a self-related questionnaire (Longo et al., 2008) showed that the factor structures of subjective response were related to the functional self, but the study was highly optimized for its own data. This data-driven approach failed to find a common factor structure for the functional self across studies. One difficulty was the lack of correspondence with studies that used different methodologies (e.g., fMRI). Therefore, a self-reported questionnaire for conceptions of the self was recently developed in a theory-driven manner. It is called the Embodied Sense of Self Scale (ESSS), and it measures three subfactors: “agency,” “ownership,” and “narrative” (Asai et al., 2016). The ESSS was developed by first listing 120 items related to the embodied sense of self, including items to assess schizotypal, depersonalizing and dissociative tendencies that relate to agency, ownership and narrative, respectively. Twenty-five items were ultimately selected for the short version, which is a reliable, valid and statistically usable scale. It significantly correlates with some related scales and clearly distinguishes healthy controls and patients with chronic schizophrenia (thought to be a disorder of the embodied sense of self).

This is the first study to examine how the subjectivity of self-related malfunctions correlates with brain structure in healthy people. For this, we searched for correlations between the ESSS subscales and measured gray matter (GM) volumes.

We focused on cortical regions that were related to the self-subscales in previous studies. We have a clear model of agency-related brain area networks because many experimental and schizophrenia patient studies have examined self-agency. These studies indicated that the cerebellum and left dorsolateral prefrontal area were involved in agency-related psychological functions. Cerebellar activation in particular was observed in subjects predicting the sensory consequences of self-action (Blakemore et al., 1999; Farrer and Frith, 2002) and those recognizing discrepancies between actual and predicted sensory consequences (Blakemore et al., 2001; Leube et al., 2003). Some studies have shown that the middle frontal gyrus detects visuomotor incongruencies (David et al., 2007; Farrer et al., 2008) and the agency of a self-propelled moving ball (Stosic et al., 2014). Schnell et al. (2007) also showed that a wide area of the middle frontal gyrus responded to the onset of visuomotor incongruence in a video game. This suggests that the dorsolateral prefrontal cortex (DLPFC) could be involved in switching the internal model of visuomotor contingency to predict body movement and sensory feedback (Imamizu et al., 2004; Imamizu and Kawato, 2008). The DLPFC is anatomically connected to the cerebellum (Kelly and Strick, 2003), which suggests that the DLPFC also has a role in switching the internal visuomotor model stored in the cerebellum in response to changing circumstances. However, structural abnormalities of the prefrontal cortex have been repeatedly reported in schizophrenic (Nickl-Jockschat et al., 2011; Schnack et al., 2014) and schizotypal individuals (Nenadic et al., 2015), while cerebellum atrophy has been less frequently reported (Zhang et al., 2015). We therefore focused on the DLPFC as an area of interest for the agency subscale.

We do not have as clear a model of the cortical networks and structural abnormalities relevant to the ownership subscale. The postcentral gyrus and insular cortex might be relevant because they are activated during the synchronous visuotactile stimulation of the rubber hand illusion (Ehrsson et al., 2004; Tsakiris et al., 2010). Additionally, the angular gyrus is a sensory association area commonly damaged in patients who frequently have out-of-body or autoscopic experiences (Blanke et al., 2004). Of these areas, the postcentral gyrus is an unlikely candidate because it is a primary somatosensory area with no reported abnormalities even in depersonalization disorder (Sierra et al., 2002), which is closely related to ownership dysfunction. The inferior parietal cortex, which contains the angular gyrus, is structurally abnormal in schizophrenic (Schnack et al., 2014) and schizotypal individuals (Nenadic et al., 2015), suggesting that it is not exclusively related to body ownership dysfunctions. However, the insular cortex is a good candidate because a body ownership-related task activates it (Tsakiris et al., 2007), but damage to this area has no impact on self-agency as measured by a task that requires distinguishing between self-generated and other-generated actions (Philippi et al., 2012). Additionally, a positron emission tomography study reported that the feeling of movement control in schizophrenia patients was related to regional cerebral blood flow in the right angular gyrus but not in the insular cortex (Farrer et al., 2004). We therefore examined the insular cortex as an area possibly correlated with the ownership subscale and irrelevant to the agency subscale.

It is difficult to identify any specific cortical region that is likely associated with the narrative self-ubscale. Araujo et al. (2015) tried to separate the core (minimal) self and autobiographical self using fMRI and showed that numerous cortical regions, including the temporal pole, precuneus and lateral occipital cortex, were involved in autobiographical self-recognition as measured with personality trait questionnaires. Legrand and Ruby (2009) showed that a task requiring self-relatedness evaluation, which is closely related to personality as an important concept of narrative self, activated cortical areas distributed over a wide cerebral network, including the medial prefrontal cortex, precuneus, temporoparietal junction and temporal poles. They suggested that this cortical network could be explained by two cognitive processes: inferential processing and memory recall. If the narrative self is a temporal expansion of the minimal self (Gallagher, 2000), it must include a process to retrieve autobiographical memory (memory recall) and a process to use these retrieved memories to generate behavioral patterns (e.g., personality) for optimizing future behavior (inferential processing). We therefore examined the network areas from Legrand and Ruby (2009) for possible correlations with the ESSS narrative self-subscale.

We hypothesized that in healthy participants regularly experiencing self anomalies in daily life, ESSS-measured subjectively reported self-related malfunctions would predict GM volume in the target cortical areas mentioned above.

## Materials and Methods

### Participants

Ninety-six healthy participants were recruited from two sites (Site A and B). Fifty-one participants (26 women and 25 men, mean age = 22.50 years, standard deviation (SD) = 3.39 years) were recruited from Site A. Forty-five participants (10 women and 35 men, mean age = 22.60 years, SD = 4.81 years) were recruited from Site B. All participants were right-handed, had no history of psychiatric or neurological disorders, and met our magnetic resonance imaging (MRI) safety criteria (e.g., not wearing any magnetic material, non-claustrophobic). Participants were paid for their participation.

This study was carried out in accordance with the recommendations of Human Research Ethics Committee of Hiroshima University and the Research Ethics Committee of Kochi University of Technology with written informed consent from all subjects. All subjects gave written informed consent in accordance with the Declaration of Helsinki. The protocol was approved by the Human Research Ethics Committee of Hiroshima University and the Research Ethics Committee of Kochi University of Technology independently.

### Questionnaires

Participants from Site A completed an 80-item version of the ESSS, while participants from Site B completed a 25-item version. Fifty-five items from the Site A ESSS were excluded, and the remaining 25 items were identical to those of the Site B ESSS. The total score and sub-scores were calculated from these 25 items.

Participants answered each item by clicking a radio button on a personal computer, with ratings on a 5-point Likert scale ranging from “Strongly disagree” to “Strongly agree”. Based on a previously reported factor analysis of this questionnaire (Asai et al., 2016), we calculated three sub-scores. The first was “ownership,” which included nine items related to malfunction of bodily awareness or body perception. The second was “narrative,” which included eights items describing the consistency of personality or self-identification. The last was “agency,” which included eight items related to the sense of controlling oneself or one’s own movement. The details of these subscales are described in Asai et al. (2016).

### MRI Data Acquisition

The participants at Site A and B both underwent MRI on a 3.0-tesla Siemens Verio Scanner (Siemens Ltd., Munich, Germany). We obtained structural MRI scans using a 32-channel head coil and whole-brain T1 weighted volumetric sequence using magnetization-prepared rapid-acquisition gradient echo (MP-RAGE). The following acquisition parameters were identical at both sites: inversion time = 900 ms, flip angle = 9°, matrix size = 256 × 256, voxel size = 1 × 1 × 1 mm, slice thickness = 1 mm, and sagittal acquisition. The Site A-specific parameters were as follows: echo time (TE) = 2.98 ms, repetition time (TR) = 2300 ms, field of view (FOV) = 256 × 256 × 176 mm, and number of slices = 176. The Site B-specific parameters were as follows: TE = 3.06 ms, TR = 2250 ms, FOV = 256 × 256 × 192 mm, and number of slices = 192.

### Preprocessing and Voxel-Based Morphometry (VBM) Analysis

Before voxel-based morphometry (VBM) analysis, all images were aligned to the anterior-posterior commissure axis to set the origin to the anterior commissure and set the images parallel to the axis. This was done using the auto_reorient.m MATLAB (MathWorks, Natick, MA, USA) script^1^, and unexpected errors were confirmed by visually inspecting the aligned images.

VBM analysis was conducted using SPM12 (rev 6225; The Welcome Department of Cognitive Neurology, London, UK) in MATLAB v. 8.3. First, all images were segmented into GM, white matter, cerebrospinal fluid, or non-brain parts after intensity non-uniformity correction. For this segmentation, we used the strong criterion (labeled “Thorough”) in SPM12 because we observed that non-brain tissue remained when we used the light criterion (“Light”). Furthermore, for anatomical normalization (affine regularization), the East Asian Brain template was selected. All other parameters were SPM12’s default settings.

Next, GM and white matter population templates were generated from all dataset images using the diffeomorphic anatomical registration through exponentiated Lie algebra (DARTEL; Ashburner, 2007). The DARTEL technique was implemented in SPM12 with default settings. First, an affine transformation was initially applied to the GM and white matter DARTEL templates to align them to the tissue probability maps in Montreal Neurological Institute space^2^. The GM images were then non-linearly warped to the DARTEL GM template in Montreal Neurological Institute space. The warped images were modulated using Jacobian determinants calculated by the nonlinear deformation field to preserve relative GM volumes even after spatial normalization. The modulated images were smoothed with an 8-mm full-width at half-maximum Gaussian kernel. The smoothed, modulated and normalized GM datasets were then statistically analyzed.

### Statistical Analysis

We performed multiple regression analysis to investigate correlations between ESSS subscale scores and regional GM volumes. For all subsequent regression analyses, the covariates included age, sex and total intracranial brain volume. The three ESSS subscales were registered as independent variables, and regional GM volume was registered as a dependent variable. To exclude any effect of site on correlations between GM volumes and ESSS scores, we made the site a dummy variable (0 = Site A, 1 = Site B) and made the dummy variable a statistical test covariate based on a suggestion made by Pardoe et al. (2008).

### Region of Interest (ROI) Analysis

For statistical VBM analysis, a mask image of the cortical region of interest (ROI) was made for each ESSS subscale. For the agency subscale, we used a mask image of Brodmann area 46 to represent the middle and inferior frontal gyri. For the ownership subscale, we used a mask image of the insular cortex. For the narrative subscale, we used a mask image of the superior medial frontal and medial orbitofrontal cortices to represent the medial prefrontal cortex, the precuneus and the angular gyrus and a mask image of the supramarginal gyrus to represent the temporoparietal junction and the middle and superior temporal poles. The mask images were generated based on Automated Anatomical Labeling and the Brodmann area separations. Statistical significance was defined as *p* < 0.05 after correction with the family-wise error (FWE) method at peak level.

### Whole-Brain Analysis

We conducted whole-brain analysis using several statistical thresholds. Based on Lieberman and Cunningham (2009), we first created a statistical map with an uncorrected *p* < 0.001 threshold and 20-voxel extent to balance Type-I and Type-II errors, but the 20-voxel extent was arbitrary and insufficiently strict for controlling Type-I errors, as shown in a study using permutation testing (Eklund et al., 2016). To confirm a statistically significant voxel extent, we calculated alternative cluster size thresholds using: (1) permutation testing of the participants’ original questionnaire scores and GM volumes; (2) the original questionnaire score sets and 96 GM volume sets randomly sampled from two open datasets (198 Beijing participants and 198 Cambridge participants) registered with the Functional Connectomes Project (Biswal et al., 2010); and (3) 96 dummy questionnaire score sets randomly generated in ranges appropriate for each scale (for example, 9–45 for the ownership subscale because it has nine 5-point scale items) and 96 GM volume sets from the same open sources used in (2). For (1), each individual’s questionnaire score set was randomly assigned to another individual’s GM volume set. For (2) and (3), 51 GM volume sets were selected from one data source and another 45 volume sets were selected from the other to imitate the original data sets coming from two different sites.

The preprocessing and statistical testing for the permuted and random sampled data were identical to those for the original data. Statistical tests were repeated 1000 times, and the 1000 maximum brain region cluster sizes that were significantly correlated with the ESSS subscales (uncorrected *p* < 0.001) were calculated and sorted in ascending order. The 950th highest value in the sorted vector was used as a statistical significance threshold for cluster size. By testing for positive or negative correlations for three subscales, we conducted six tests and generated six cluster size significance thresholds for each repetition. The maximum value among these six thresholds was finally adopted as the statistical analysis threshold.

## Results

Averages and SDs of ESSS total and subscale scores are listed in Table 1, and total brain volumes are listed in Table 2.

**Table 1:** **Average ESSS total and subscale scores**.

	ESSS agency	ESSS ownership	ESSS narrative	ESSS total
Average	22.40	16.75	24.56	63.71
SD	5.79	5.88	5.91	15.00

*Abbreviations: ESSS, Embodied Sense of Self Scale; SD, standard deviation*.

**Table 2:** **Average volumes and SDs of gray matter, white matter and total brain**.

	Gray matter	White matter	Total brain
Average (cm^3^)	773.09	464.86	1237.95
SD (cm^3^)	58.23	48.59	97.69

*Abbreviations: SD, standard deviation*.

### ROI Analysis

**Figure 1:**
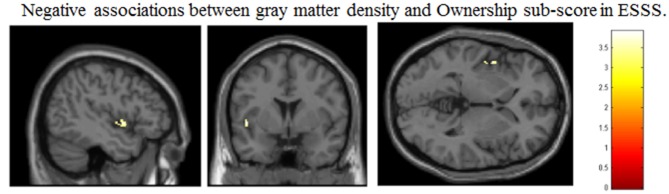
**Significant correlations between GM volumes and ESSS ownership subscale scores by ROI analysis**. Abbreviations: GM, gray matter; ESSS, Embodied Sense of Self Scale; ROI, region of interest.

Correlation analysis revealed a significant negative correlation between ownership subscale scores and GM volumes in the left posterior insular cortex (peak coordinates: *x* = −47, *y* = 2, *z* = −2; number of voxels = 42; *t* = 3.91, *p* < 0.05 after FWE correction at peak level; Figure 1). There were no other significant correlations between regional GM volumes and ESSS subscale scores.

### Whole-Brain Analysis

Permutation testing of the original data calculated 615 as the minimum significant cluster size. Random sampling tests that paired open source cortical structure data with either the original questionnaire scores or randomly generated questionnaire scores calculated significance thresholds of 797 or 682, respectively.

With an uncorrected *p* < 0.001 threshold and a 20-voxel extent, all significant correlations between GM volumes and questionnaire scores are listed in Table 3. As listed in Table 3, no cortical area had a cluster size greater than the lowest statistical threshold (615 voxles). The greatest cluster size which was found in analysis with our original data was 224 for the positive correlation between the narrative subscale scores and GM volumes in the left inferior temporal gyrus. There were therefore no significant correlations in whole-brain analysis using corrected cluster size criteria.

**Table 3:** **Brain regions in which local GM volume was significantly correlated with ESSS subscale scores**.

Location name	*x*	*y*	*z*	*k*	*t*
*Negative correlations between GM volumes and ownership scores*
L insula (BA 13)	−47	2	−2	134	3.91
L angular gyrus (BA 39)	−53	−56	30	31	3.48
R postcentral gyrus (BA 2)	59	−21	47	32	3.37
*Positive correlations between GM volumes and narrative scores*
L lingual gyrus (BA 18)	−35	−92	−20	96	3.89
L inferior temporal gyrus	−47	−57	−11	224	3.85
R cuneus (BA 18)	9	−101	15	60	3.80
L superior temporal pole (BA 22)	−50	9	−2	107	3.80
L precuneus (BA 5)	−11	−39	56	46	3.37
*Positive correlation between GM volumes and agency scores*
R cerebellum crus 1	44	−59	−36	90	3.46
*Negative correlations between GM volumes and agency scores*
L medial orbitofrontal cortex (BA 11)	−15	38	−24	68	3.72
L middle frontal cortex (BA 9)	−30	35	26	60	3.69

*Regions significantly correlated with each ESSS subscale are listed. The codes in parentheses indicate Brodmann areas (e.g., BA13 = Brodmann area 13). In the first row, x, y and z refer to Montreal Neurological Institute coordinates, k refers to the number of voxels in each significant area and t refers to the t-score in each brain region (local maxima). Abbreviations: GM, gray matter; ESSS, Embodied Sense of Self Scale; L, left; R, right*.

The significant correlations between ESSS subscales and GM volumes in whole-brain analysis with an uncorrected *p* < 0.001 threshold and a 20-voxel extent were described below.

#### Correlation between Agency Subscale Scores and GM Volumes

**Figure 2:**
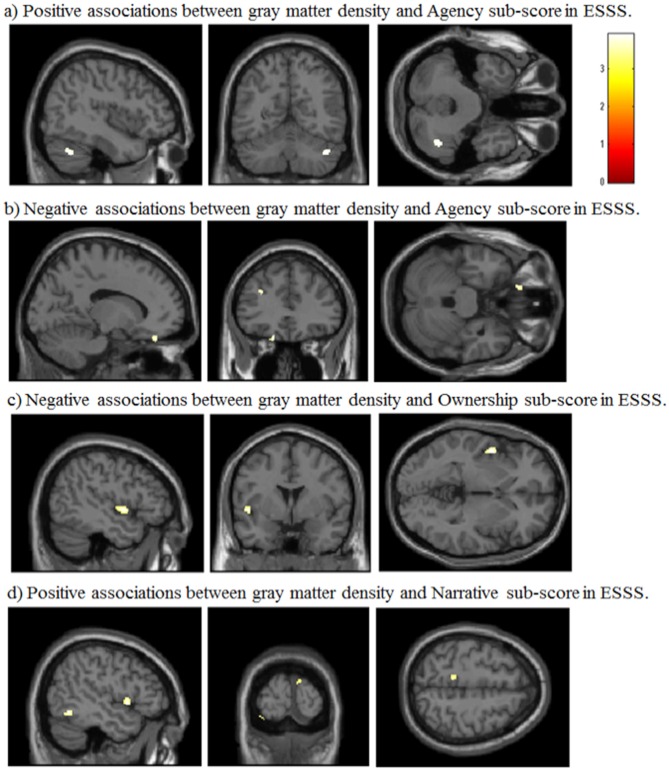
**Significant correlations between GM volumes and ESSS subscale scores by whole-brain analysis. (A)** Positive correlations between GM volumes and ESSS agency subscale scores. The right cerebellum is highlighted in the sagittal, coronal and transverse views [44, −59 −36]. **(B)** Negative correlations between GM volumes and ESSS agency subscale scores. The left medial orbitofrontal cortex is focused in the sagittal, transverse view. The left medial orbitofrontal and left medial frontal cortices are highlighted in the coronal view [−14, 34 −27]. **(C)** Negative correlations between GM volumes and ESSS ownership subscale scores. The left insular cortex is focused in the sagittal, coronal and transverse views [−47, 2, −2]. **(D)** Positive correlations between GM volumes and ESSS narrative subscale scores. The left superior temporal pole and left inferior temporal gyrus are highlighted in the sagittal view. The left lingual gyrus and right cuneus are highlighted in the coronal view. The left precuneus is highlighted in the transverse view [−47, −96, 56]. Abbreviations: GM, gray matter; ESSS, Embodied Sense of Self Scale.

Agency subscale scores were positively correlated with GM volumes in the right cerebellum (peak coordinates: *x* = 44, *y* = −59, *z* = −36; number of voxels = 90; *t* = 3.46; Figure 2A). Agency scores were negatively correlated with GM volumes in two cortical areas: the left medial orbitofrontal cortex (peak coordinates: *x* = −15, *y* = 38, *z* = −24; number of voxels = 68; *t* = 3.72; Figure 2B) and the left medial frontal cortex (peak coordinates: *x* = −30, *y* = 35, *z* = 26; number of voxels = 60; *t* = 3.69).

#### Correlation between Ownership Subscale Scores and GM Volumes

Ownership subscale scores were negatively correlated with GM volumes in three brain areas: the left insular cortex (peak coordinates: *x* = −47, *y* = 2, *z* = −2; number of voxels = 134; *t* = 3.91; Figure 2C), left angular gyrus (peak coordinates: *x* = −53, *y* = −56, *z* = 30; number of voxels = 31; *t* = 3.48), and right postcentral gyrus (peak coordinates: *x* = 59, *y* = −21, *z* = 47; number of voxels = 32; *t* = 3.37). Ownership scores were not significantly positively correlated with GM volumes in any examined area.

#### Correlation between Narrative Subscale Scores and GM Volumes

Narrative subscale scores were positively correlated with GM volumes in five cortical areas: the left lingual gyrus (peak coordinates: *x* = −35, *y* = −92, *z* = −20; number of voxels = 96; *t* = 3.89), left inferior temporal gyrus (peak coordinates: *x* = −47, *y* = −57, *z* = −11; number of voxels = 224; *t* = 3.85; Figure 2D), right cuneus (peak coordinates: *x* = 9, *y* = −101, *z* = 15; number of voxels = 60; *t* = 3.80), left superior temporal pole (peak coordinates: *x* = −50, *y* = 9, *z* = −2; number of voxels = 107; *t* = 3.80), and left precuneus (peak coordinates: *x* = −11, *y* = −39, *z* = 56; number of voxels = 46; *t* = 3.37). Narrative scores were not significantly negatively correlated with GM volumes in any examined area.

## Discussion

We aimed to determine the relationship between subjectively reported self-related malfunction and GM volume. Self-related malfunctions were subjectively measured using our recently developed ESSS questionnaire (Asai et al., 2016). The ESSS measures daily experiences rather than illusory feelings induced by specific experimental tasks (e.g., the rubber hand illusion). ROI analysis showed that ownership subscale scores were negatively correlated with left posterior insula GM volumes. This association suggests that daily experiences of self-related malfunctions could induce cortical structure changes. We also conducted whole-brain analysis, but this showed no significant correlations between cortical areas and ESSS subscale scores.

### Correlations between the Left Posterior Insula and the Ownership Subscale

As expected, we observed a significant correlation between ownership subscale scores and left posterior insula GM volumes. The insular cortex has been repeatedly shown to be related to body ownership through such tests as the rubber hand illusion (Tsakiris et al., 2007; Limanowski et al., 2014), but it is not strictly limited to body ownership because agency-related tasks can also activate it (Leube et al., 2003). Additionally, both the right and left insular cortices are activated by viewing a video consistently subject to self-controlled movement (Farrer and Frith, 2002; Farrer et al., 2004). Given that some lesion studies have shown that the bilateral insular cortex is not responsible for self agency (Philippi et al., 2012; Damasio et al., 2013), the insular cortex is not solely related to agency or ownership but includes additional complex cognitive functions. Indeed, Kurth et al. (2010) conducted a meta-analysis of the insular cortex’s psychological functions and showed their differentiation into emotional, chemosensory, sensorimotor and cognitive domains. From these, interoception in the sensorimotor domain had the location (−41, 2, 3 for the left hemisphere) closest to that of the left insular cortex in our results (−47, 2, −2). The insular cortex is also activated by such tasks as listening to one’s own heartbeat or suppressing the urge to void, consistent with Seth’s model in which the insular cortex is related to interoceptive inference and self-embodiment (Seth, 2013). This suggests that the insular cortex might be reduced in size by impaired self-awareness of body ownership due to altered interoceptive inference.

However, the ESSS ownership subscale includes the following item: “Sometimes it feels like my body is jerky like a robot”. The term “jerky” in this sentence could mean uncontrollable movement, suggesting that altered body sensation is closely related to movement related malfunctions (for all the items, see Asai et al., 2016). This suggests that the ownership subscale is not fully separated from the agency domain. Altogether, it remains unclear whether the two minimal self factors, agency and ownership, are sufficiently separated in the ESSS. Future studies should directly investigate this.

### Correlations of GM Volume with ESSS Subscale Scores

Whole-brain analysis based on the criteria by Lieberman and Cunningham (2009) revealed that ESSS subscale scores were significantly correlated with GM volumes in some areas, and these regions were highly predictable based on the findings from previous studies. For example, agency subscale scores were correlated with GM volumes in the cerebellum and middle frontal gyrus, which were activated during active movement inducing a sense of agency over a rubber hand (Tsakiris et al., 2010). Ownership subscale scores were correlated with GM volumes in the postcentral gyrus, insula and angular gyrus, which might be engaged in bodily self-consciousness, including body ownership (Blanke et al., 2004; Ehrsson et al., 2004; Tsakiris et al., 2010). We found that narrative subscale scores were positively correlated with GM volumes in the left lingual gyrus, the left inferior temporal gyrus, the right cuneus, the left superior temporal pole and the left precuneus. These areas overlapped with the network activated by a self-relatedness evaluation task (Legrand and Ruby, 2009). However, these correlations could not survive under strict criteria using permutation testing, which suggests that they are not statistically robust.

### Limitations

One limitation of this study is that scanning was conducted at two sites. Although almost all scan parameters were identical, this might have contaminated the results, as might other uncontrolled variables such as region, culture and experimenter. The two sites were located in different prefectures on different islands, so the cultural differences could be sufficient to affect the results. Additionally, the scanning method may have differed between experimenters (e.g., fixation of the head or the degree of detail given in instructions), which could have affected the structural image. We attempted to control for site effects by following a recommendation in Pardoe et al. (2008). Some studies (e.g., Moorhead et al., 2009) have shown that a VBM study’s statistical power can be improved by adjusting probability maps for the distribution of gray and white matter. Since this requires at least two scans on each scanner, we could not apply it to our results, so we should consider the possibility that important relationships between brain areas and questionnaire scores may have gone undetected.

One major limitation of our study is that no significant correlations between regional GM volumes and ESSS subscale scores were found in cluster size analysis. A recent study cautioned that statistical significance thresholds using cluster size tend to cause 60%–80% Type I error rates (Eklund et al., 2016). This can be mitigated by using permutation testing (Eklund et al., 2016), but we found that this led to a significance threshold of more than 600 voxels, far greater than the highest observed value at 224 voxels. Consequently, our whole-brain analysis showed no significant correlations between ESSS subscales and GM volumes. The significance threshold was little different even if we used open source human brain structure data from the Functional Connectomes Project (Biswal et al., 2010) that were also used in Eklund et al. (2016). This analysis assumes that there is no relationship between ESSS scores and GM volumes, so we expected a low significance threshold. However, the minimum significant cluster size was 797, which was even higher than that obtained with our own data. To further generalize this criterion, we also conducted the same repetition test using the same open source GM volume data but with dummy questionnaire data generated with a score range restriction. This analysis too calculated a significance threshold of more than 600 voxels. Altogether, these findings suggest that when analyzing correlations between GM volumes and questionnaire scores in a relatively small organ like the cortex, it might not be appropriate to use cluster size as a criterion, at least if the ESSS is the questionnaire.

## Conclusion

Collectively, ESSS-measured, ownership-related self malfunctions in daily life were confirmed to be associated with the insular cortex. This is consistent with previous findings about the cortical areas related to self ownership. It also shows that the ESSS can be a quick assessment tool to predict individual differences in cortical volume related to ownership malfunction.

## Author Contributions

NK and TA designed the study. NK, TA, KM, RK, TU, TY, and HK performed the experiment. NK and TN analyzed the data. NK, TA, and TN wrote the manuscript. SY supervised the project. All authors approved the final version of the manuscript.

## Funding

This study was supported by Research Fellowships for Young Scientists, grant numbers 26780418 and 16H05958 (Japan Society for the Promotion of Science). The Japan Science and Technology Agency’s Center of Innovation Program partially supported this research.

## Conflict of Interest Statement

The authors declare that the research was conducted in the absence of any commercial or financial relationships that could be construed as a potential conflict of interest.

## Footnotes

^1^http://www.nemotos.net/?p=17

^2^http://www.mni.mcgill.ca/

## Abbreviations

DARTEL: diffeomorphic anatomical registration through exponentiated Lie algebra
DLPFC: dorsolateral prefrontal cortex
ESSS: Embodied Sense of Self Scale
GM: gray matter
VBM: voxel-based morphometry.
